# Introducing IOS_11_ as an extended interactive version of the ‘Inclusion of Other in the Self’ scale to estimate relationship closeness

**DOI:** 10.1038/s41598-024-58042-6

**Published:** 2024-04-17

**Authors:** Malte Baader, Chris Starmer, Fabio Tufano, Simon Gächter

**Affiliations:** 1 Department of Finance, University of Zurich, Zurich, Switzerland; 2 Present Address: School of Economics, University of Nottingham, Nottingham, England; 3 School of Business, University of Leicester, Leicester, England

**Keywords:** Psychology, Human behaviour

## Abstract

The study of relationship closeness has a long history in psychology and is currently expanding across the social sciences, including economics. Estimating relationship closeness requires appropriate tools. Here, we introduce and test a tool for estimating relationship closeness: ‘IOS_11_’. The IOS_11_ scale has an 11-point response scale and is a refinement of the widely used Inclusion-of-Other-in-the-Self scale. Our tool has three key features. First, the IOS_11_ scale is easy to understand and administer. Second, we provide a portable, interactive interface for the IOS_11_ scale, which can be used in lab and online studies. Third, and crucially, based on within-participant correlations of 751 individuals, we demonstrate strong validity of the IOS_11_ scale in terms of representing features of relationships captured by a range of more complex survey instruments. Based on these correlations we find that the IOS_11_ scale outperforms the IOS scale and performs as well as the related Oneness scale.

## Introduction

Relationships are a central element of human sociality. Here, we present and test a tool designed to estimate the subjectively perceived *quality* of a relationship between two agents (“relationship closeness”). Extensive literatures study the determinants of relationship closeness and investigate its impact on wide-ranging dimensions of human well-being including health, the incidence and resolution of conflict, and economic productivity^1,2^. Based on existing research, the study of relationship closeness can offer important insights into the human condition and contribute to public understanding of pressing contemporary issues such as how to build healthier, more resilient, productive, and inclusive societies^3,4^. Our current contribution is to introduce an improved technique for measuring relationship closeness that is low-cost to implement and well-suited to a wide range of applications.

Influential work in psychology dating back several decades has developed a range of techniques for quantifying relationship closeness. Prominent examples include: the *Relationship Closeness Inventory* (RCI)^5^, the *Subjective Closeness Index (SCI)*^5^, the *Love and Liking scale* (LLS)^6^ as well as the *Personal Acquaintance Measure (PAM)*^7^. While these methods focus on different types or aspects of relationships and differ in their conceptual foundations, they share the common feature that their implementation requires responses to, sometimes quite extensive, multi-item questionnaires.

Our primary concern is with an offshoot from this literature, which has sought to develop more compact tools for measuring relationship closeness that can serve as valid substitutes for extensive multi-item questionnaires. Two well established and highly cited tools are the *Inclusion of Other in the Self* (*IOS*) scale^8^ and the *Oneness* scale^9^ which we describe in detail in the next section. Both techniques are well-known and the two key papers that introduced and popularized them had, at the time of writing, accumulated almost 9000 citations between them^8,9^, with only a minority of papers citing both articles. Both tools are quick and easy to implement and have been shown to accurately estimate relationship closeness as measured by extensive survey instruments^5–7^. This holds across a wide range of relationship classes, from acquaintances to close friends^10^. The tools have been widely used across the social and behavioral sciences especially in the disciplines of psychology and sociology^11–16^ and in various applied fields such as health^17–19^; there is also growing interest in new areas of application (e.g., research in economics^20–22^ or computer science^23,24^) where, until recently, these tools had barely been used at all.

To date, however, researchers considering using one of these tools have faced a tradeoff. Specifically, the IOS scale is more “convenient” to implement (it requires measurement of just one scale instead of two) but comparative testing has shown that the Oneness scale is the more “predictive” tool in that it correlates more strongly with other, more complex, measures of relationship closeness as found by Gächter et al.^10^. Since its publication^10^, several studies^25–32^ have relied on their evidence to motivate the use of the IOS scale as a good predictor of relationship closeness even though it is not the best available tool in this respect. While sacrificing accuracy for simplicity or convenience may have been a defensible trade-off, as we demonstrate below, it is no longer necessary.

In this paper, we propose an estimation instrument which builds closely on the original IOS scale. A key feature is that we extend the tools’ response range (from a 7-point) to an 11-point scale. Based on this feature, we refer to our tool as the “IOS_11_ scale”. The primary motivation for extending the response range is that it provides a more nuanced measurement tool, with its degree of granularity more comparable to that of the two-item Oneness scale. To see why, consider a participant who responds with scores of, say, 3 and 4 on the two Oneness items. This participant receives a score of 3.5, a value not measurable on the original IOS scale. If the advantage of Oneness derives from this finer implied scale, the expanded IOS_11_ scale should substantially close that gap. We do not presume that finer granularity is the only plausible explanation of the differential performance between the IOS scale and the Oneness scale, however. Other contending possibilities, for example, are that the two items of the Oneness scale pick up somewhat distinct aspects of relationship closeness or that two-item estimation is inherently less noisy^33,34^. We address the former possibility further in the Results section. While our data shed some light on what factors may be at play, our primary objective was to test the conjecture that finer granularity might reduce the gap between the predictive performance of the Oneness scale and our IOS_11_ scale.

Minded by the important growth of, often very large-scale, data collection in online environments^35,36^, a second innovative feature of the IOS_11_ scale is that we implement it via an interactive, computerized, interface. The result is a simple and intuitive task suited to a range of computerized environments from lab to online participant pools such as Amazon MTurk or Prolific.

Following Gächter et al.^10^, we test the performance of the IOS_11_ scale by examining its correlation with a set of other well-established but more elaborate estimates of relationship closeness (RCI^5^, SCI^5^*,* LLS^6^, and PAM^7^) and we benchmark the performance of our tool against Oneness and the original IOS scale. The IOS_11_ scale thereby complements other work developing the IOS scale to suit an online study environment^37–39^. We also include a pre-registered replication of Gächter et al.’s^10^ Study 3 alongside our validation of the IOS_11_ scale. We find that the IOS_11_ scale elicits relationship closeness more accurately than the IOS scale and just as well as the more complex Oneness scale. We argue that our tool with its combination of high accuracy and cost-effectiveness is an attractive new approach for fast, convenient, and effective estimation of relationship closeness.

## Methods

### The IOS_11_ scale

**Figure 1:**
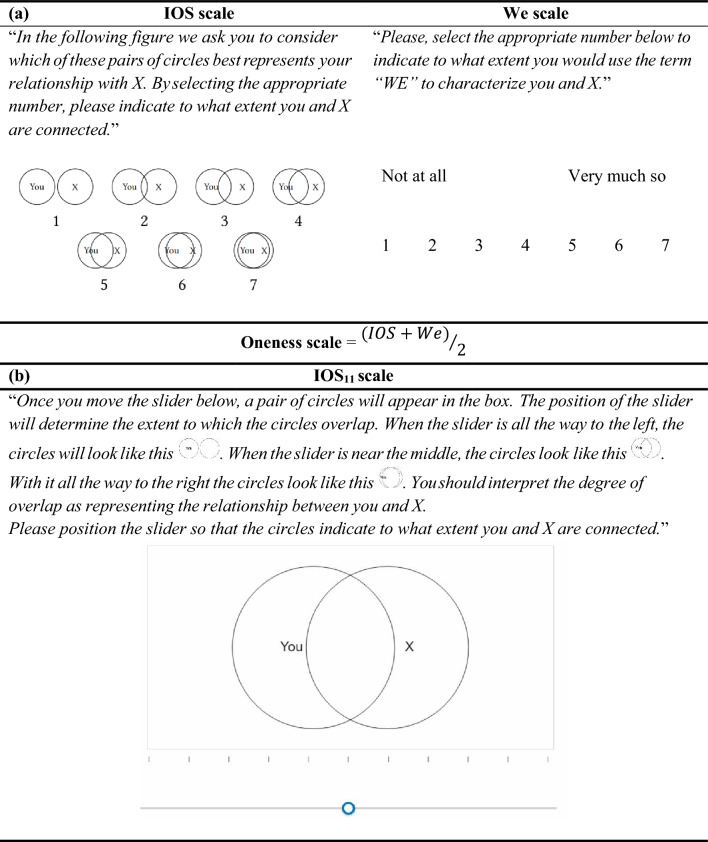
Graphical comparison of the interfaces of the IOS scale and our IOS_11_ scale. Panel (**a**) depicts the IOS scale, the We scale, and the Oneness scale. Panel (**b**) illustrates the IOS_11_ scale. The initial screen participants see when entering the elicitation is blank. For illustration purposes, we are depicting the slider at a central position in this figure.

The left hand side of panel (a) in Fig. 1 presents the original IOS scale^8^. A respondent is required to say which of the seven pairs of circles best represents their relationship with another identified individual. As noted in the introduction, responses to this simple task correlate (Spearman’s $\rho \in [$0.514, 0.820], *p* < 0.001) with estimates based on considerably more complex measurement approaches^10^. However, the Oneness scale, which takes the average of responses on two items—the IOS scale and the We scale^40^ (top right of Fig. 1)—has been shown to outperform the basic IOS scale in its correlation with other estimates of relationship closeness^10^.

In developing the IOS_11_ scale, panel (b), and for reasons already explained, we conjectured that extending the 7-point response scale of the original IOS scale might enhance its predictive accuracy. Extending the number of pairs of circles from which participants can choose, however, creates two obvious challenges. The first is how to visualize an increased number of overlapping circles without their presentation becoming too cluttered, complicated, or confusing. Secondly, we needed to decide by how many options the answer range should be extended.

We addressed the first of these challenges by developing our tool as a computerized version of the IOS scale using an interactive screen that allows participants to intuitively adjust the degree to which circles overlap. Our layout is displayed in the bottom panel (b) of Fig. 1. Participants move a slider below the circle diagram to adjust the degree to which the circles overlap. These changes to the scale do not affect the portability, ease of explanation, or the time it takes to complete the task compared to the original IOS task. The resulting tool also has the obvious attraction that the IOS_11_ scale can be implemented in a wide range of computerized environments supporting easy use in online surveys and online or lab experiments (it can be accessed under 10.17605/OSF.IO/9DBR6).

This leads us to the second consideration of how many degrees of overlap to offer. The move to a computerized environment allows, in principle, the implementation of a very fine-grained (quasi-continuous) scale.

However, some authors have suggested that using a continuous or ‘visual-analogue’ scale can be a source of noise if respondents “*[are] unable to reliably make meaningful and valid fine-grained distinctions”*^41^. Moved by this consideration, we stick with a discrete version of the task. To enhance comparability to previous studies, we kept the maximum and minimum overlap of circles identical to the IOS scale. We then chose the number of levels such that the change in distance between the centers of the circles is approximately linear and so that the original IOS levels form a subset of the extended version (see online Appendix A.2 for details). This leads to a setup with 11 relationship closeness levels as shown in the middle column of Table 1. The left-hand column of Table 1 shows how scores on the original IOS scale map into a subset of scores on the new tool. Additionally, the rightmost column of Table 1 shows how the IOS_11_ scale can be recoded to a 7-point scale with endpoints matching the original IOS scale for comparability.

**Table 1:** Comparison of the IOS and IOS11 Scales.

	IOS scale	IOS_11_ scale	IOS_11_ scale
			Recoded
	1	1	1
		2	1.5
	2	3	2
		4	2.5
	3	5	3
	4	6	4
	5	7	5
		8	5.5
	6	9	6
		10	6.5
	7	11	7

Columns 1–3 show the IOS scale, the IOS_11_ scale, and a recoded version of the IOS_11_ scale, respectively. ‘X’ serves as a placeholder for the initial of the person considered. The original scale does not reduce the distance between circles linearly. Thus, we extend our scale in the range [1,2,3] and [5,6,7] to yield an almost linear change in overlap. ‘IOS_11_ scale recoded’ is a re-coding of the ‘IOS_11_ scale’ that retains the 1–7 scale.

### Procedures

We test convergent validity of the IOS_11_ scale by examining how well it correlates with scores obtained through a range of other measures of relationship closeness and we benchmark its performance against the original IOS scale and the Oneness scale. We employ a between-participant design, where each participant either performs the two tasks necessary to estimate Oneness (i.e., the average responses on the IOS and We scales) or completes our IOS_11_ task. We then explore the within-participant correlation of scores from each of the IOS, Oneness, and IOS_11_ scales to a series of well-established survey instruments designed to capture relationship closeness. As noted above, the different scales that we use are the *RCI*^5^, the* SCI*^5^, the *LLS*^6^ as well as the *PAM*^7^.

Note that some of these measures were constructed to capture different specific degrees of relationships (e.g., the RCI^5^ explicitly refers to romantic relationships, whereas the PAM^7^ was designed for acquaintances). However, from a behavioral scientist’s perspective, it is useful to have a general-purpose and portable measurement tool that can be reliably used in a range of relationships. For that reason, following Gächter et al.^10^, we employ a between-subject variation where participants were asked to either consider a very close person; a friend; or an acquaintance across all of the core questions within the study. Hence, our main experiment can be considered a two-by-three treatment design varying Oneness and IOS_11_ tasks on the one hand and the type of relationship considered on the other. Since we borrow from Gächter et al.^10^ when testing the validity of the IOS_11_ scale, our hypotheses as well as the statistical analyses closely follow their work.

We presented the instruments eliciting relationship closeness in random order, followed by questions regarding demographics and other individual attributes. Further, to ensure salience of the considered person throughout the study, we ask participants in the beginning of the experiment to provide the initials of the person they are thinking of. These initials are then inserted in all parts where the instructions explicitly refer to another person. We also asked each participant to rate a stranger via either the Oneness scale or the IOS_11_ scale to examine individual-level variation in interpretation of the scale. This showed limited evidence of any consistent demographic determinants (see online Appendix A.1). The full instructions and details of the various measures of relationship closeness employed as benchmarks are in the online Appendix B.

We pre-registered our study (https://www.socialscienceregistry.org/trials/7947) and collected data online in July 2021 using the survey software Qualtrics^42^. Our pre-registration includes a description of the experimental design, the targeted sample size as well as the key variables of interest. Although the pre-registration did not set out a detailed plan for data analyses, as our approach replicates and extends Gächter et al.^10^ we follow their statistical analyses. The study was approved by the Nottingham School of Economics’ Research Ethics Committee.

**Figure 2:**
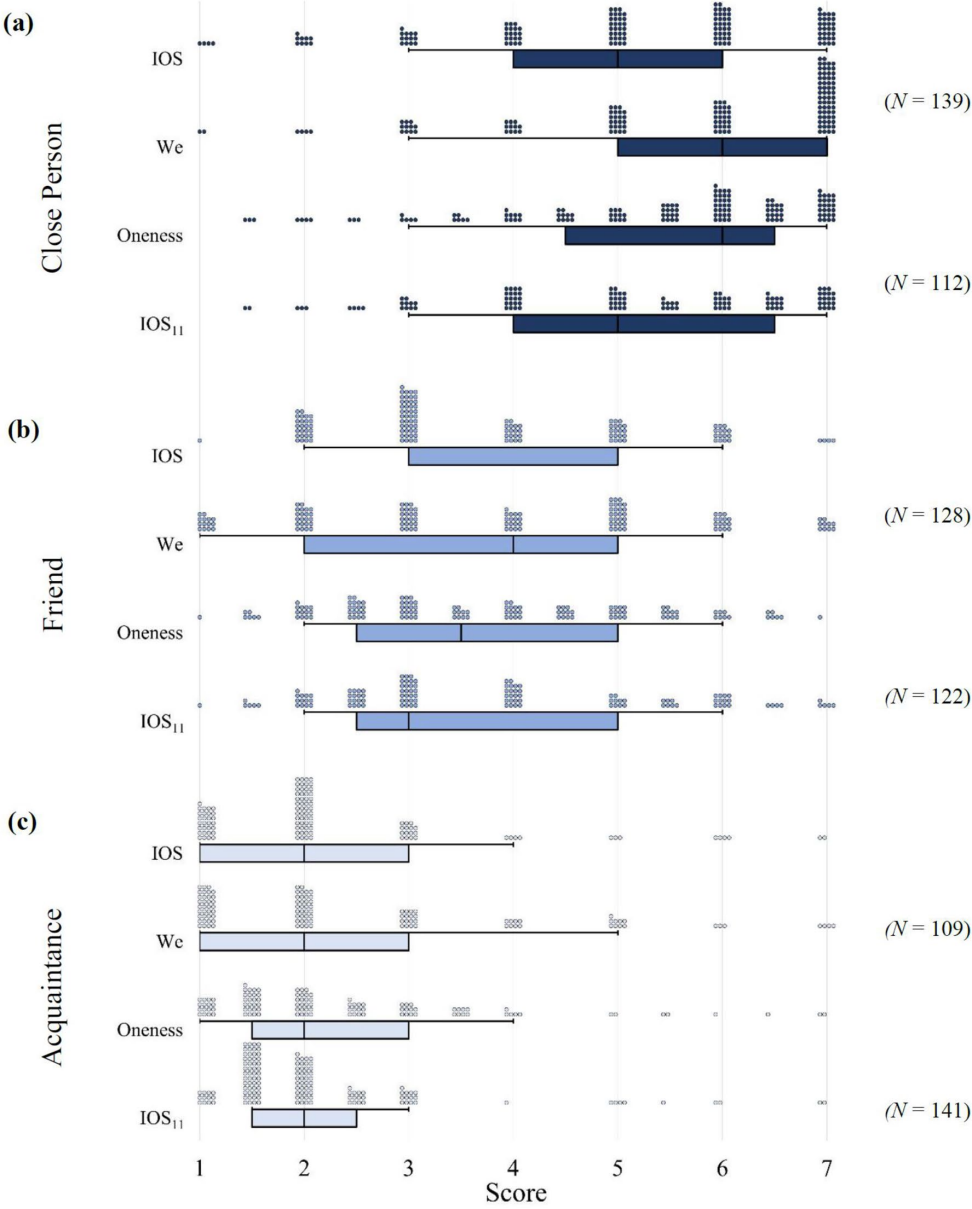
Relationship levels, elicitation tools and recorded scores. In each panel, we present scores of the IOS scale, the We scale, the Oneness scale and the IOS_11_ scale from top to bottom. The Oneness scale is the arithmetic mean of responses on the IOS and We scale. The IOS_11_ scores are recoded as defined in Table 2. The boxplots capture the median and the interquartile range. The whiskers range from the 10*th* to 90*th* percentile. Each circle in the distribution plot captures a unique observation. Different relationship levels are presented in three distinct panels. (**a**) Close person; (**b**) friend; (**c**) acquaintance.

We recruited 751 participants with *N* ≈ 125 per treatment using Prolific’s UK sample (the exact numbers of participants in each treatment are in Fig. 2). All participants completed an informed consent form at the start of the study and all methods in this study were conducted in accordance with relevant guidelines for the ethical treatment of human participants. The mean age of our participants is 35.22 years (*SD* = 13.86, *Mdn* = 32, *Min* = 17, *Max* = 75) with 501 (67%) identifying as female, 242 (32%) identifying as male, and 10 participants not revealing their gender. The sample includes 29% students and 56% of the participants are either in full- or part-time employment. Using an online participant pool such as Prolific therefore provided us with a more heterogeneous demographic than utilizing a student sample. We also obtained additional survey data of other demographics directly from Prolific including age, gender, education levels, and details about the participant’s household. We paid a flat fee of £1.20 per participant and the study took about 15 minutes to complete.

## Results

As a first descriptive benchmarking of the IOS_11_ scale against the IOS and Oneness scales, we examine the reported relationship closeness scores across different treatments. All analyses below utilize the recoded scores for the IOS_11_ scale (as per final column of Table 1) to allow for direct comparisons between methodologies. However, our results are also robust when using the IOS_11_ scale without recoding the scores.

Figure 2 plots scores of the IOS and We scale, the Oneness scale (the arithmetic mean of responses on the IOS and We scale), and the IOS_11_ scale for each level of relationship. The box plots capture the interquartile range for each estimate and the underlying distributions are indicated by the circles above the boxes. The different colors indicate whether the person thought of was a close person (dark blue), a friend (blue) or an acquaintance (light blue). The different scales (IOS, We, Oneness, and IOS_11_) for each relationship level are then presented in separate bars from top (close person, panel a) to bottom (acquaintance, panel c).

Figure 2 shows that for all four instruments, there is clear and coherent variation in reported closeness comparing different relationship levels. Based on pairwise Kolmogorov–Smirnov (KS) tests, participants who considered a close person reported significantly higher scores than those who considered a friend (*D*_*IOS*_ = 0.379; *D*_*We*_ = 0.440; *D*_*Oneness*_ = 0.446; *D*_*IOS11*_ = 0.340; *p* < 0.001) and scores for those considering an acquaintance were lower still (*D*_*IOS*_ = 0.532; *D*_*We*_ = 0.352; *D*_*Oneness*_ = 0.431; *D*_*IOS11*_ = 0.589; *p* < 0.001). Moreover, the figure also shows that reported levels of closeness are similar across methods. Notwithstanding this general coherence, Fig. 2 reveals some differences across the distributions of scores for different methods, in the comparison of IOS and We scale scores.

Notice that for ratings of a close person, the interquartile range and median value for the We scale lie to the right of that for the IOS scale reflecting, in part, a markedly stronger tendency for participants to record maximum values on the We scale, relative to the IOS scale (*D* = 0.237; *p* = 0.001 for KS test comparing the two distributions). This is suggestive evidence that IOS and We scales may, to some extent, be capturing different aspects of relationship closeness and, if they are, this could be part of the explanation for why the Oneness scale, which combines the two scales, has tended to psychometrically outperform the IOS scale alone. Notice, however, that relative to the IOS scale, at the eyeball level the distribution of the IOS_11_ scale more closely resembles the distribution of the Oneness scale.

Based on KS tests, the IOS_11_ and Oneness scores are statistically indistinguishable from each other for a close person (*D* = 0.142; *p* = 0.164); a friend (*D* = 0.063; *p* = 0.966); and an acquaintance (*D* = 0.158; *p* = 0.095). To the extent that the Oneness scale outperforms the IOS scale in tracking other estimates of relationship closeness, these results suggest the possibility that the IOS_11_ scale might close some of that performance gap.

Table 2 reports within-participant Spearman’s rank correlations between IOS, Oneness and IOS_11_ (columns) and a set of nine benchmark scores obtained from distinct scales (rows) with darker shades of blue indicating stronger correlations. Columns 1 to 3 display the results for the IOS scale, the Oneness scale, and the IOS_11_ scale from our study, whereas columns 4 and 5 reproduce results for the IOS scale and the Oneness scale from Gächter et al.^10^ for comparison. The first row reports correlations with the overall RCI benchmark score and the next three rows report correlations with its three sub-components (frequency, diversity, and strength)^5^. “Love” and “Like” scores are two elements of LLS^6^. The final row reports correlations with an Index of relationship closeness (IRC); this is a single index developed by Gächter et al.^10^ but derived from the set of other benchmark scores^5–7^ using a principal components analysis^10^.

**Table 2:**
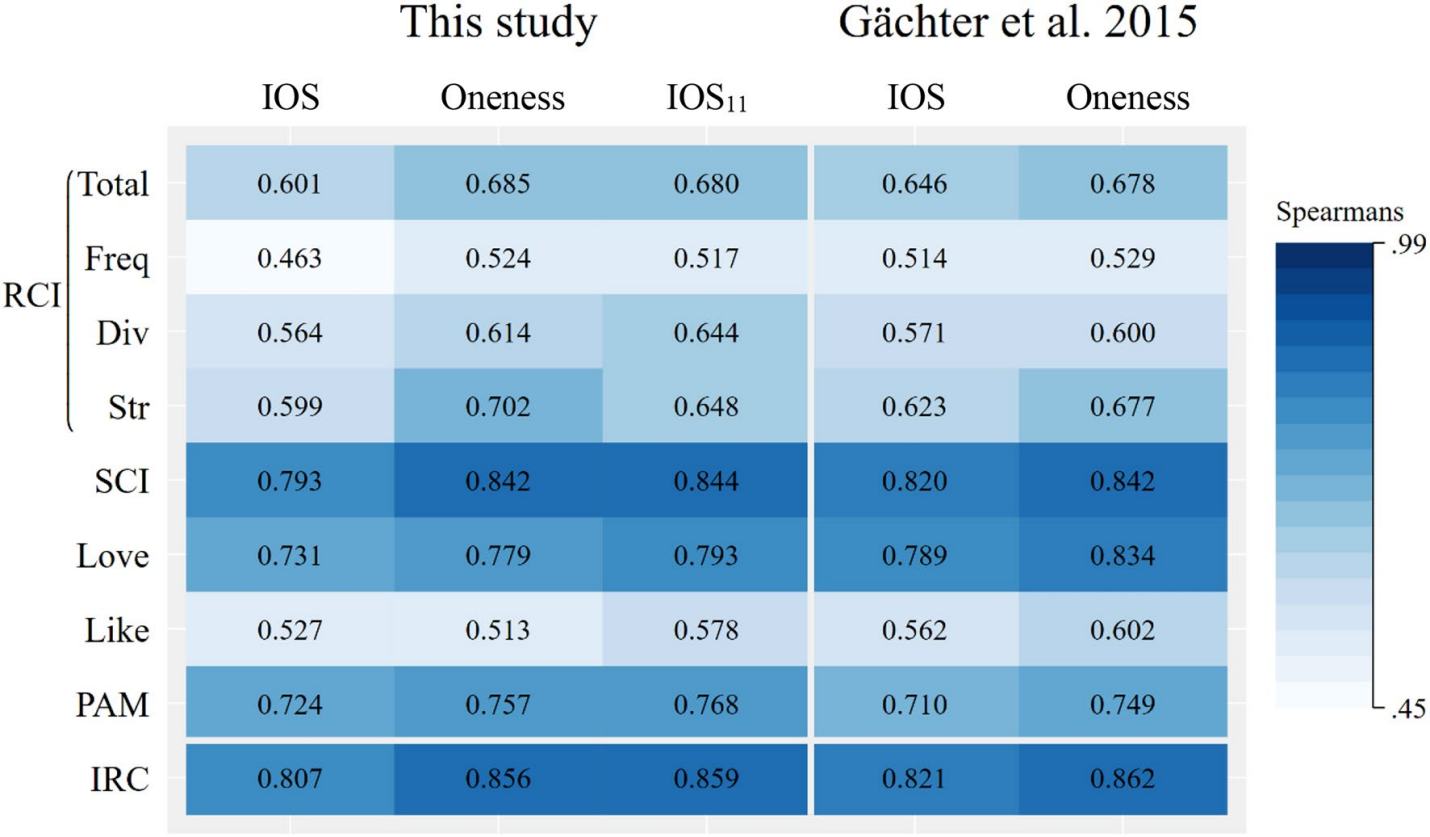
Correlations across scores obtained by relationship scales.

Columns 1–3 display results from this study, columns 4–5 results from Gächter et al.^10^. All cells in the table present Spearman’s rank correlations, all are significant at the 1% level. Scores of benchmark scales are in the rows and the estimates of relationship closeness in the different columns. RCI is the *Relationship Closeness Inventory* with its subdomains *Frequency*, *Diversity* and *Strength*^5^. SCI indicates the *Subjective Closeness Index*^5^*,* the *Love* and *Liking* scales are from Rubin^6^*.* PAM refers to the *Personal Acquaintance Measure*^7^ and IRC to the *Index of Relationship Closeness.*

Across the table, we find moderately strong to strong correlations throughout; all are statistically significant at the 1% level. Table 3 reports pairwise tests of differences between correlation coefficients (IOS scale vs. Oneness scale; IOS scale vs. IOS_11_ scale; IOS_11_ scale vs. Oneness scale).

**Table 3:** Pairwise comparisons of correlation coefficients.

	IOS vs. Oneness	IOS vs. IOS_11_	Oneness vs. IOS_11_
Benchmark scale			
RCI total	− 1.882 (0.059)	− 1.760 (0.078)	0.133 (0.894)
RCI frequency	− 1.072 (0.284)	− 0.941 (0.346)	0.137 (0.890)
RCI diversity	− 1.005 (0.315)	− 1.657 (0.097)	− 0.645 (0.518)
RCI strength	− 2.343 (0.019)	− 1.068 (0.285)	1.289 (0.197)
SCI	− 1.920 (0.054)	− 2.023 (0.043)	− 0.092 (0.926)
Love	− 1.475 (0.140)	− 1.966 (0.049)	− 0.482 (0.630)
Like	0.260 (0.795)	− 0.961 (0.336)	− 1.222 (0.221)
PAM	− 0.943 (0.345)	− 1.298 (0.194)	− 0.350 (0.726)
IRC	− 2.085 (0.037)	− 2.252 (0.024)	− 0.155 (0.876)

*z*-statistics (with *p*-values in parentheses) of a test of equality of correlation coefficients described in Cohen et al.^43^ The table rows correspond with Table 2 by presenting benchmark scales. The three columns, respectively, present results for comparisons of: IOS scale versus Oneness scale; IOS scale versus IOS_11_ scale and Oneness scale versus IOS_11_ scale. RCI is the *Relationship Closeness Inventory* with its subdomains *Frequency*, *Diversity* and *Strength*^5^. SCI is the *Subjective Closeness Index*^5^*,* the next two rows are the *Love* and *Liking scales*^6^*.* PAM is the *Personal Acquaintance Measure*^7^ and IRC is the *Index of Relationship Closeness.*

Table 2, combined with the tests presented in Table 3, reveals three broad patterns. First, correlations between Oneness and the various benchmark scores tend to be systematically higher than those between the benchmarks and the original IOS scale (in Table 3, comparing the IOS scale with the Oneness scale, there are two cases where the correlation is significantly higher for the Oneness scale, at the 5% level or higher, and none in the opposite direction). Second, the IOS_11_ scale outperforms the original IOS scale (in Table 3, there are three cases where the IOS_11_ scale has a significantly higher correlation with a comparator benchmark, at the 5% level or better, and no cases where IOS performs better). Thirdly, we find no significant differences when comparing the correlations between the Oneness scale and the IOS_11_ scale for each of the nine benchmark scales (in Table 2, across the nine benchmarks, differences go in both directions, but they are never significantly different at the 5% level and few of the *p*-values in the final column of Table 3 are close to significance at any conventional level).

The three broad patterns just identified each hold for the IRC: this is meaningful because the IRC is arguably the most informative of the benchmarks (by virtue of being the principal component of the larger set of estimates). More specifically, based on results reported in the final row of Table 3, we replicate the finding of Gächter et al.^10^ that the Oneness scale outperforms the IOS scale in terms of its correlation with the IRC (*z* = − 2.085; *p* = 0.037 in Table 3); we see that the correlation of the IOS_11_ scale with the IRC is stronger than that for the original IOS scale (*z* = − 2.252; *p* = 0.024); and it is statistically indistinguishable from the Oneness scale (*z* = − 0.155; *p* = 0.876). Since scores three to five are identical in the IOS and the IOS_11_ scale, we replicate Table 3 by excluding participants with these scores. We find that all our results are robust (see online Appendix A.3).

It is also worth noting that, overall, we replicate the evidence from Gächter et al.^10^ in finding correlation coefficients that very closely mimic the original results. This is noteworthy as we utilized a different study population (US vs. UK) on different platforms (MTurk vs. Prolific), and a substantive amount of time has passed since the original data collection (2014 vs. 2021).

Based on these results, we summarize our main finding as follows: In terms of convergent validity, our tool, the IOS_11_ scale, matches the performance of the Oneness scale in terms of its correlation with a set of scores obtained through established estimates of relationship closeness, but it does so whilst maintaining the simplicity of the single-item IOS scale.

## Discussion and conclusion

In this paper, we have introduced the IOS_11_ scale as a tool for eliciting relationship closeness. The primary advantage of the IOS_11_ scale lies in addressing the issue that, until now, researchers considering using IOS-like scales have faced a tradeoff between the simplicity of the single-item IOS scale and the added accuracy of the two-item Oneness scale. The IOS_11_ scale resolves this tension by offering a new 11-point version of the IOS scale which, according to our results, is statistically indistinguishable from the Oneness scale in terms of its ability to track a range of more complex questionnaire-based estimates of relationship closeness^5–7^. For those considering the use of some IOS-style tool, the IOS_11_ scale provides a convenient, highly portable, and efficient method for the elicitation of relationship closeness in any computerized environment.

Our study also complements ongoing research developing estimation techniques for relationship closeness^37–39^. Two of these studies develop online versions of the IOS scale using a continuous scale and, like us, conjecture that a more fine-grained tool may increase precision^38,39^. A third study compares scores obtained from the standard IOS scale with a continuous version and a step-choice version^37^. Using a within-participant design, the authors conclude that a continuous version is least likely to suffer from a *no overlap* bias, where participants avoid selecting the pair of circles without overlap. However, none of the three papers benchmark to the Oneness scale or the RCI^5^, SCI^5^*,* LLS^6^, or PAM^7^.

Previous studies utilizing scales from the IOS family have also investigated other psychometric properties, such as test–retest reliability, convergent validity, and predictive validity. In the original paper that introduced the IOS scale^8^, the authors find a high correlation (*r* = 0.83) across a two-week test–retest, and strong evidence of convergent validity (0.09 ≤ *r* ≤ 0.45 with other estimates of relationship closeness) and discriminant validity (*r* = 0.09 with a methodologically similar, but conceptually unrelated measure)^8^. Similarly, also for the Oneness scale, previous work found strong evidence of test–retest reliability (*r* = 0.93)^21^ across two-weeks and convergent validity (0.36 ≤ Spearman’s* ρ* ≤ 0.58) with other estimates of relationship closeness^10^. Whilst we find clear similarities between the estimates of relationship closeness as revealed by the IOS_11_ and IOS scales and the Oneness scale, in terms of their correlations with other estimates of relationship closeness, future work could usefully explore other psychometric properties of the IOS_11_ scale including test–retest reliability, discriminant validity, convergence of self- and partner-report or its validity in predicting other meaningful behavior.

### Supplementary Information

Supplementary Information.

## Data Availability

The data, the IOS_11_ software, and the analysis files are available via the Open Science Framework (10.17605/OSF.IO/9DBR6). The experiments were pre-registered at the American Economic Association's registry for randomized controlled trials (Registration number AEARCTR-0007947; see https://www.socialscienceregistry.org/trials/7947).
