# Supplementary material for: Introducing IOS11 as an extended interactive version of the ‘Inclusion of Other in the Self’ scale to estimate relationship closeness
Source: Sci Rep. 2024 Apr 17;14:8901. doi: 10.1038/s41598-024-58042-6 (PMC11024120; doi:10.1038/s41598-024-58042-6)
Supplement: Supplementary file 1 — Supplementary Information. [file 41598_2024_58042_MOESM1_ESM.pdf]

Online Appendix for
Introducing IOS_{11} as an extended interactive version of the 'Inclusion of Other in the Self' scale to estimate relationship closeness

Malte Baader[1], Chris Starmer[2], Fabio Tufano[3] & Simon Gächter[2]

[1]Department of Banking and Finance, University of Zurich

[2]School of Economics, University of Nottingham

[3]School of Business, University of Leicester

11 April 2024

Contents

Appendix A – Additional Analyses 2

Appendix A.1 – Demographics and responses 2

Appendix A.2 – Linearity 4

Appendix B – Experimental instructions 6

Appendix A – Additional Analyses

Table A1

		Close Person				Friend				Acquaintance			
		We	IOS	Oneness	IOS_{11}	We	IOS	Oneness	IOS_{11}	We	IOS	Oneness	IOS_{11}
Close P.	We	-											
	IOS	0.001	-										
	Oneness	0.000	0.022	-									
	IOS_{11}	0.000	0.855	0.164	-								
Friend	We	0.000	0.000	0.000	0.000	-							
	IOS	0.000	0.000	0.000	0.000	0.428	-						
	Oneness	0.000	0.000	0.000	0.000	0.270	0.343	-					
	IOS_{11}	0.000	0.000	0.000	0.000	0.279	0.806	0966	-				
Acquaint.	We	0.000	0.000	0.000	0.000	0.000	0.000	0.000	0.000	-			
	IOS	0.000	0.000	0.000	0.000	0.000	0.000	0.000	0.000	0.524	-		
	Oneness	0.000	0.000	0.000	0.000	0.000	0.000	0.000	0.000	0.073	0.141	-	
	IOS_{11}	0.000	0.000	0.000	0.000	0.000	0.000	0.000	0.000	0.002	0.026	0.095	-

Comparison of response distributions across scales and relationship levels. The table reports results of pairwise Kolmogorov-Smirnov tests between different relationship levels and measurement instruments. We report p-values, indicating the statistical likelihood that two distributions are significantly different from each other.

Appendix A.1 – Demographics and responses

To explore individual-level variation in interpretation of the scale we also elicited relationship closeness towards a stranger. Thus, by abstracting from a familiar individual, we extrude information of a baseline relationship closeness that a person feels towards another. In addition, for each participant, we also elicited the Big Five (Donnellan et al., 2006), survey measures of charitable giving (Falk et al., 2022) and the Balanced Inventory of Desirable Reporting (BIDR) (Paulhus & Reid, 1991). Since we are ultimately interested in gaining information into the IOS_{11}, we conduct this analysis for Oneness as well as IOS_{11} and explore whether any observed heterogeneity in responses is robust across methodologies. Utilizing a multivariate ordered probit regression with relationship closeness scores, Oneness and IOS_{11}, as the dependent variables presented in Table A.3, we find that across methodologies female participants provide half a point lower score in relationship closeness (p-value < 0.05). In addition, participants willing to donate higher amounts to charity also report higher scores to strangers. This holds across tools, indicating no unintended heterogeneity in responses using the IOS_{11}.

Table A2

	Oneness	IOS_{11}
Age	-0.00309	-0.00336
	(0.00775)	(0.00813)
Female	-0.428**	-0.429**
	(0.184)	(0.172)
Student	0.104	0.0560
	(0.243)	(0.228)
Employment status (Reference: Not in paid work)		
Unemployed	0.0395	0.134
	(0.321)	(0.316)
Due to start	0.197	0.102
	(0.502)	(0.605)
Part-Time	0.177	0.0495
	(0.287)	(0.260)
Full-Time	-0.0222	-0.115
	(0.280)	(0.252)
Other	0.0870	0.396
	(0.382)	(0.349)
Education (Reference: No qualifications)		
Secondary (e.g. GED/GCSE)	-0.378	3.973
	(0.608)	(226.6)
High school/A-levels	-0.141	4.391
	(0.579)	(226.6)
College	-0.688	4.488
	(0.617)	(226.6)
Undergraduate	-0.678	4.246
	(0.580)	(226.6)
Graduate	-0.233	4.114
	(0.604)	(226.6)
Doctorate	0.464	-0.870
	(0.970)	(309.1)
Politics (Reference: Left)		
Center	-0.222	-0.0810
	(0.190)	(0.172)
Right	0.0457	-0.479
	(0.258)	(0.304)
Household		
# of siblings	-0.0689	-0.0559
	(0.0629)	(0.0738)
# of household	-0.000972	-0.0462
	(0.0646)	(0.0610)
Autistic spectrum (Reference: No)		
In process	0.631	-3.890
	(0.624)	(321.1)
Yes (adult)	-5.033	-0.812
	(150.3)	(0.935)
Yes (child)	1.172*	-6.154
	(0.640)	(321.1)
No but identify	0.209	-0.579
	0.631	-3.890
Charitable giving		
Giving good cause	(0.410)	(0.382)
	-0.0382	0.0628*
Donate charity	(0.0312)	(0.0358)
	0.000620*	0.000731**
Big Five		
Extraversion	0.0305	0.0189
	(0.0214)	(0.0209)
Agreeableness	0.0413	-0.00455
	(0.0311)	(0.0283)
Conscientiousness	-0.0182	0.00209
	(0.0251)	(0.0232)
Neuroticism	-0.0416	-0.00226
	(0.0273)	(0.0251)
Openness	0.00757	-0.0843**
	(0.0343)	(0.0359)
Desirable Reporting		
BIDR	0.0126	-0.0150
	(0.0171)	(0.0153)
Observations	239	245
Pseudo R^2	0.0642	0.0747

Regressions results of individual characteristics on relationship closeness to a stranger. The table reports regression results ordered probit regressions with Oneness and IOS_{11} for a stranger as the dependent variable. Demographics are either collected within the study or obtained through Prolific.

* $p < 0.05$, ** $p < 0.01$, *** $p < 0.001$

Table A3

		This study			Gächter et al. 2015	
		IOS	Oneness	IOS11	IOS	Oneness
RCI	Total	0.612	0.685	0.695	0.646	0.678
	Freq	0.472	0.524	0.530	0.514	0.529
	Div	0.568	0.614	0.658	0.571	0.600
	Str	0.616	0.702	0.677	0.623	0.677
SCI		0.805	0.842	0.854	0.820	0.842
Love		0.754	0.779	0.808	0.789	0.834
Like		0.555	0.513	0.611	0.562	0.602
PAM		0.739	0.757	0.783	0.710	0.749
IRC		0.826	0.856	0.869	0.821	0.862

Spearmans: .99 – .45

Table A3 excludes pariticpants that responded with 4 in the IOS or IOS_{11}. Columns 1-3 display results from this study, columns 4-5 results from Gächter, et al.[10] All cells in the table present Spearman's rank correlations, all are significant at the 1% level. Scores of benchmark scales are in the rows and the measures of relationship closeness in the different columns. RCI is the *Relationship Closeness Inventory* with its subdomains *Frequency*, *Diversity* and *Strength*. SCI indicates the *Subjective Closeness Index (SCI)*, Love the *Love scale* and *Like the Liking scale*. PAM refers to the *Personal Acquaintance Measure* and IRC to the *Index of Relationship Closeness*.

Appendix A.2 – Linearity

Figure A1:

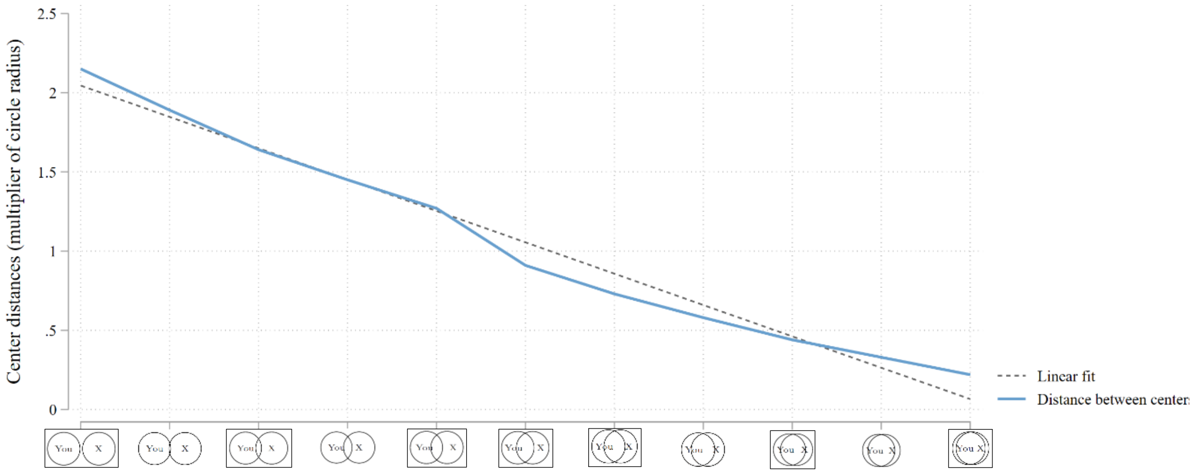

Distance between centers of circles. The y-axis captures the distance between the centers of the pair of circles when the radius = 1. The exact values are [2.15, 1.89, 1.64, 1.45, 1.27, 0.91, 0.73, 0.58, 0.44, 0.33, 0.22]. When the radius increases, the distance between circles needs to be increased proportionally. The pair of circles with a border indicate circles used in the original IOS tool.

Appendix B – Experimental instructions

[Instructions were encountered by all subjects except when stated otherwise. Here, we report the full set of questions we used. The questionnaire was implemented using the survey software Qualtrics (www.qualtrics.com) In the implementation, except for the introductory/background questions, we randomised the order of the other blocks that measure relationship closeness (IOS, WE, SCI, PAM, RCI, Love, and Like). [X] represents the named initial in all questions. All programmes are available upon request.]

The questions relate to several scales, for an overview:

- Q1-Q6 and Q155-Q156 are introductory/background questions
- Q7 refers to the "Inclusion of the Other in the Self" (IOS) scale (Aron et al., 1992) OR the IOS_{11} scale
- Q8 refers to the "We" scale (Cialdini et al., 1997)
- Q9 and Q10 refer to the "Subjective Closeness Index" (SCI) (Berscheid et al., 1989)
- Q11-Q25 refer to the "Personal Acquaintance Measure" (PAM) (Starzyk et al., 2006)
- Q26-Q64 refer to the "Relationship Closeness Inventory" (RCI) (Berscheid et al., 1989)
- Q65-Q77 and Q78-Q90 refer respectively to Loving and Liking scales (Rubin, 1970)
- Q91-Q130 refer to the "Balanced Inventory of Desirable Reporting" (BIDR) (Paulhus & Reid, 1991)
- Q131-Q150 refer to the "Mini-IPIP scales: Measure of the Big 5" (Mini IPIP) (Donnellan et al., 2006)
- Q151-Q152 refer to "Self-reported altruism" (Falk et al., 2018)
- Q153 refers to the "Inclusion of the Other in the Self" (IOS) scale (Aron et al., 1992) OR the IOS_{11} scale for a stranger
- Q154 refers to the "We" scale (Cialdini et al., 1997) for a stranger

----

Thank you for participating in our Study!

In this study we will ask you to respond to a questionnaire on the nature of interpersonal relationships.

Our interest is entirely scientific. All answers will be treated confidentially and will only be reported in aggregated statistical form.

There are no right or wrong answers in this survey, we are only interested in your honest assessment. If you feel uncomfortable answering some questions you will have opportunities to select "prefer not to answer" as an answer.

Please enter your Prolific ID here: **[Text input]**

We are currently investigating the nature of interpersonal relationships. As part of this study, we would like you to answer the following questions about your relationship with another person.

[Depending on the 'relationship level' treatment subjects saw one of the following]

Specifically, we would like you to choose the one person with whom you have the closest, deepest, most involved, and most intimate relationship, and answer the following questions with regard to this particular person. For some of you, this person may be a dating partner or someone with whom you have a romantic relationship. For others of you, this person may be a close, personal friend, family member, or companion. It makes no difference exactly who this person is as long as she or he is the one person with whom you have the closest, deepest, most involved, and most intimate relationship.

OR

Specifically, we would like you to choose a person with whom you have a good friendship, who is more than an acquaintance, but not your closest, or most intimate relationship, and answer the following questions with regard to this particular person. For some of you, this person may be a personal friend. For others of you, this person may be a family member, or companion. It makes no difference exactly who this person is as long as she or he is a good friend, who is more than an acquaintance, but not your closest, or most intimate relationship.

OR

Specifically, we would like you to choose a person whom you consider an acquaintance, but no more than an acquaintance, and answer the following questions with regard to this particular person. For some of you, this person may be a colleague at work. For others of you, this person may be a neighbour, or member of your wider social network. It makes no difference exactly who this person is as long as she or he is a person who you consider an acquaintance, but no more than an acquaintance.

Please select this person carefully since this decision will affect the rest of this study. With this person in mind, please respond to the following questions.

[Q1] Who is this person? Please give the initial of the first name only. **[Text entry]**

This person will be referred to as [X] in all questions that follow.

[Q2] What is your gender?

- Female
- Male
- Prefer not to say

[Q3] What is [X]'s gender?

- Female
- Male
- Prefer not to say

[Q4] What is your age? **[Numeric input]**

[Q5] How long have you known [X]? Please indicate the number of years and months.

- Years **[Numeric input]**
- Months **[Numeric input]**

[Q6] Which of the following best describes your relationship with [X]? (Check only one)

- WORK: co-worker
- WORK: your boss/supervisor
- WORK: your subordinate
- FAMILY: aunt/uncle

- FAMILY: sister/brother
- FAMILY: parent
- FAMILY: cousin
- ROMANTIC: married
- ROMANTIC: engaged
- ROMANTIC: living together
- ROMANTIC: dating only this person
- ROMANTIC: dating this person and others
- FRIEND: close friend (non-romantic)
- FRIEND: casual friend
- ACQUAINTANCE (please give short description)
- OTHER (please give short description)
- prefer not to say

[Depending on the treatment allocation subjects saw <u>one</u> of the following two IOS scales]

[Q7a] In the following figure we ask you to consider which of these pairs of circles best describes your relationship with this person (referred to as [X] in all questions that follow). In the figure "X" serves as a placeholder for X, that is, you should think of "X" being [X]. By selecting the appropriate number please indicate to what extent you and [X] are connected. **[Numeric input]**

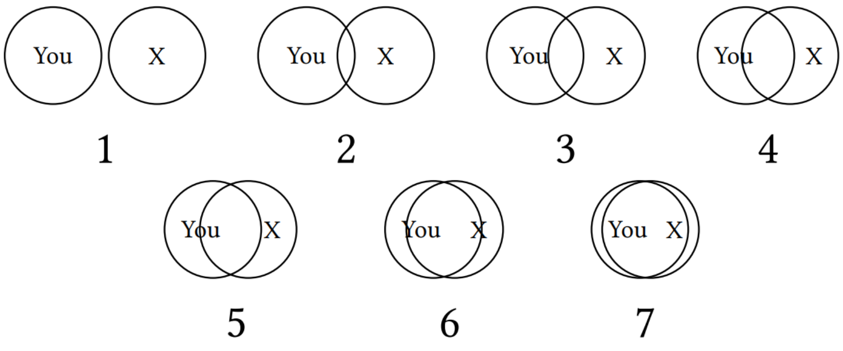

OR

"Once you move the slider below, a pair of circles will appear in the box. The position of the slider will determine the extent to which the circles overlap. When the slider is all the

way to the left, the circles will look like this . When the slider is near the middle, the circles look like this . With it all the way to the right the circles look like this . You should interpret the degree of overlap as representing the relationship between you and X.

Please position the slider so that the circles indicate to what extent you and X are connected."

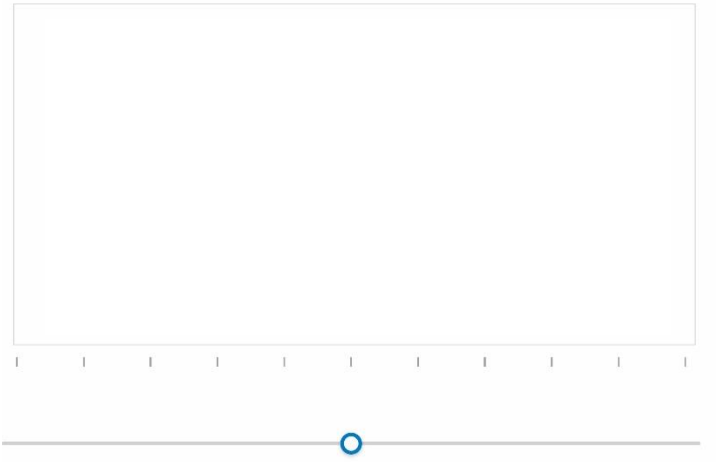

[Q8] Please, select the appropriate number below to indicate to what extent you would use the term "WE" to characterize you and [X]. **[8-item Likert scale; 1 - not at all; 7 - very much so; 8 - prefer not to answer]**

[Q9] Relative to all your other relationships (both same and opposite sex) how would you characterize your relationship with [X]? **[8-item Likert scale; 1 - not close at all; 7 - very close; 8 - prefer not to answer]**

[Q10] Relative to what you know about other people's close relationships, how would you characterize your relationship with [X]? **[8-item Likert scale; 1 - Not close at all; 7 - very close; 8 - prefer not to answer]**

Please think of [X], read each statement carefully, and click the answer that best corresponds to your agreement or disagreement with each statement. **[For Q11 - Q25: 6-item Likert scale; 1 - Strongly Disagree; 2 - Disagree; 3 - Neither Agree nor Disagree; 4 - Agree; 5 - Strongly Agree; 6 - Prefer Not to Answer]**

[Q11] I have known [X] for many years.

[Q12] I have known [X] for a long time.

[Q13] I have gone to parties (social events) with [X].

[Q14] [X] often hides his/her true feelings from me.

[Q15] Seeing [X] is part of my weekly routine.

[Q16] I know what [X]'s goals are.

[Q17] [X] hides his/her true feelings from me.

[Q18] [X] has told me about his/her interests.

[Q19] I have spent time with [X] and his/her friends.

[Q20] [X] avoids showing his/her true feelings around me.

[Q21] [X] and I go way back.

[Q22] I am familiar with [X]'s friends.

[Q23] I see [X] a lot.

[Q24] Seeing [X] is part of my daily routine.

[Q25] [X] has told me what his/her goals are.

We would like you to estimate the amount of time you typically spend alone with [X] during the day. We would like you to make these time estimates by breaking the day into morning, afternoon, and evening, although you should interpret each of these time periods in terms of your own typical daily schedule. (For example, if you work a night shift, "morning" may actually reflect time in the afternoon, but is nevertheless time immediately after waking.) Think back over the past week and select the average amount of time, per day, that you spent alone with [X], with no one else around, during each time period. If you did not spend any time with [X] in some time periods, select 0 hour(s) and 0 minutes.

[Q26] DURING THE PAST WEEK, what is the average amount of time, per day, that you spent alone with [X] in the MORNING (e.g., between the time you wake and 12 noon)?

- Hours **[Numeric input]**
- Minutes **[Numeric input]**

[Q27] DURING THE PAST WEEK, what is the average amount of time, per day, that you spent alone with [X] in the AFTERNOON (e.g., between 12 noon and 6pm)?

- Hours **[Numeric input]**
- Minutes **[Numeric input]**

[Q28] DURING THE PAST WEEK, what is the average amount of time, per day, that you spent alone with [X] in the EVENING (e.g., between 6pm and bedtime)?

- Hours **[Numeric input]**
- Minutes **[Numeric input]**

[Q29] Compared with the "normal" amount you usually spend alone with [X], how typical was the past week?

- typical
- not typical

[Q30] The following is a list of different activities that people may engage in over the course of one week. For each of the activities listed, please check all of those that you have engaged in alone with [X] in the past week. Check only those activities that were done alone with [X] and not done with [X] in the presence of others.

In the past week, I did the following activities alone with [X] (Check all that apply)

- did laundry
- prepared a meal
- watched TV
- went to an auction/antique show
- attended a non-class lecture or presentation
- went to a restaurant
- went to a grocery store
- went for a walk/drive
- discussed things of a personal nature
- went to a museum/art show
- planned a party/social event
- attended class
- went on a trip (e.g., vacation or weekend)

- cleaned house/apartment
- went to church/religious function
- worked on homework
- spent time together on the internet (e.g., Skype, FaceTime, surfing together, etc)
- discussed things of a non-personal nature
- went to a clothing store
- talked on the phone
- went to a movie
- ate a meal
- participated in a sporting activity
- outdoor recreation (e.g., sailing)
- went to a play
- went to a bar
- visited family
- visited friends
- went to a department, book, hardware store, etc.
- played cards/board game
- attended a sporting event
- exercise (e.g., jogging, aerobics)
- went on an outing (e.g., picnic, beach, zoo, winter carnival)
- wilderness activity (e.g., hunting, hiking, fishing)
- went to a concert
- went dancing
- went to a party
- played music/sang
- other (please describe briefly)

The following questions concern the amount of influence [X] has on your thoughts, feelings, and behavior. Using the 7-point scale below, please indicate the extent to which you agree or disagree (from 1 - I strongly disagree to 7 - I strongly agree) **[For Q31 - Q57: 8-item Likert scale; 1 - I strongly disagree; 7 - I strongly agree; 8 - prefer not to answer]**

[Q31] [X] will influence my future financial security.

[Q32] [X] does not influence everyday things in my life.

[Q33] [X] influences important things in my life.

[Q34] [X] influences which parties and other social events I attend.

[Q35] [X] influences the extent to which I accept responsibilities in our relationship.

[Q36] [X] does not influence how much time I spend doing household work.

[Q37] [X] does not influence how I choose to spend my money.

[Q38] [X] influences the way I feel about myself.

[Q39] [X] does not influence my moods.

[Q40] [X] influences the basic values that I hold.

[Q41] [X] does not influence the opinions that I have of other important people in my life.

[Q42] [X] does not influence when I see, and the amount of time I spend with, my family.

[Q43] [X] influences when I see, and the amount of time I spend with, my friends.

[Q44] [X] does not influence which of my friends I see.

[Q45] [X] does not influence the type of career I have.

[Q46] [X] influences or will influence how much time I devote to my career.

[Q47] [X] does not influence my chance of getting a good job in the future.

[Q48] [X] influences the way I feel about the future.

[Q49] [X] does not have the capacity to influence how I act in various situations.

[Q50] [X] influences and contributes to my overall happiness.

[Q51] [X] does not influence my present financial security.

[Q52] [X] influences how I spend my free time.

[Q53] [X] influences when I see [X] and the amount of time the two of us spend together.

[Q54] [X] does not influence how I dress.

[Q55] [X] influences how I decorate my home (e.g., apartment, house, dorm room, ...).

[Q56] [X] does not influence where I live.

[Q57] [X] influences what I watch on TV

Now we would like you to tell us how much [X] affects your future plans and goals. Using the 7-point scale below, please indicate the degree to which your future plans and goals are affected by [X] by clicking the appropriate scale. If an area does not apply to you (e.g., because you have no plans or goals in that area), click "1 - not at all". **[For Q58 - Q64: 8-item Likert scale; 1 - not at all; 7 - a great extent; 8 - prefer not to answer]**

[Q58] [X] affects my vacation plans.

[Q59] [X] affects my marriage plans.

[Q60] [X] affects my plans to have children.

[Q61] [X] affects my plans to make major investments (house, car, etc.).

[Q62] [X] affects my plans to join a club, social organization, church, etc.

[Q63] [X] affects my school-related plans.

[Q64] [X] affects my plans for achieving a particular financial standard of living.

Please, read each statement carefully, and click the answer that best corresponds to your agreement or disagreement with each statement. **[For Q65 - Q77: 10-item Likert scale; 1 - Not at all true; disagree completely; 5 - Neither agree nor disagree; 9 - Definitely true; agree completely; 10 - Prefer not to answer]**

[Q65] If [X] were feeling badly, my first duty would be to cheer him/her up.

[Q66] I feel that I can confide in [X] about virtually everything.

[Q67] I find it easy to ignore [X]'s faults.

[Q68] I would do almost anything for [X].

[Q69] I feel very possessive toward [X].

[Q70] If I could never be with [X] I would feel miserable.

[Q71] If I were lonely my first thought would be to seek [X] out.

[Q72] One of my primary concerns is [X]'s welfare.

[Q73] I would forgive [X] for practically anything.

[Q74] I feel responsible for [X]'s well-being.

[Q75] When I am with [X] I spend a good deal of time just looking at him (her).

[Q76] I would greatly enjoy being confided in by [X].

[Q77] I would be hard for me to get along without [X].

Please, read each statement carefully, and click the answer that best corresponds to your agreement or disagreement with each statement. **[For Q78 - Q90: 10-item Likert scale; 1 - Not at all true; disagree completely; 5 - Neither agree nor disagree; 9 - Definitely true; agree completely; 10 - Prefer not to answer]**

[Q78] When I am with [X] we are almost always in the same mood.

[Q79] I think that [X] is unusually well adjusted.

[Q80] I would highly recommend [X] for a responsible job.

[Q81] In my opinion, [X] is an exceptionally mature person.

[Q82] I have great confidence in [X]'s good judgment.

[Q83] Most people would react very favorably to [X] after a brief acquaintance.

[Q84] I think that [X] and I are quite similar to each other.

[Q85] I would vote for [X] in a group election.

[Q86] I think that [X] is one of those people who quickly wins respect.

[Q87] I feel that [X] is an extremely intelligent person.

[Q88] [X] is one of the most likable people I know.

[Q89] [X] is the sort of person whom I myself would like to be.

[Q90] It seems to me that it is very easy for [X] to gain admiration.

Using the scale as a guide, select a number beside each statement to indicate how much you agree with it. **[For Q91 - Q130: 8-item Likert scale; 1 - not true; 4 - somewhat true; 7 - very true; 8 - prefer not to answer]**

[Q91] My first impressions of people usually turn out to be right.

[Q92] It would be hard for me to break any of my bad habits.

[Q93] I don’t care to know what other people really think of me.

[Q94] I have not always been honest with myself.

[Q95] I always know why I like things.

[Q96] When my emotions are aroused, it biases my thinking.

[Q97] Once I’ve made up my mind, other people can seldom change my opinion.

[Q98] I am not a safe driver when I exceed the speed limit.

[Q99] I am fully in control of my own fate.

[Q100] It’s hard for me to shut off a disturbing thought.

[Q101] I never regret my decisions.

[Q102] I sometimes lose out on things because I can’t make up my mind soon enough.

[Q103] The reason I vote is because my vote can make a difference.

[Q104] My parents were not always fair when they punished me.

[Q105] I am a completely rational person.

[Q106] I rarely appreciate criticism.

[Q107] I am very confident of my judgments.

[Q108] I have sometimes doubted my ability as a lover.

[Q109] It’s all right with me if some people happen to dislike me.

[Q110] I don’t always know the reasons why I do the things I do.

[Q111] I sometimes tell lies if I have to.

[Q112] I never cover up my mistakes.

[Q113] There have been occasions when I have taken advantage of someone.

[Q114] I never swear.

[Q115] I sometimes try to get even rather than forgive and forget.

[Q116] I always obey laws, even if I’m unlikely to get caught.

[Q117] I have said something bad about a friend behind his or her back.

[Q118] When I hear people talking privately, I avoid listening.

[Q119] I have received too much change from a salesperson without telling him or her.

[Q120] I always declare everything at customs.

[Q121] When I was young I sometimes stole things.

[Q122] I have never dropped litter on the street.

[Q123] I sometimes drive faster than the speed limit.

[Q124] I never read sexy books or magazines.

[Q125] I have done things that I don’t tell other people about.

[Q126] I never take things that don’t belong to me.

[Q127] I have taken sick-leave from work or school even though I wasn’t really sick.

[Q128] I have never damaged a library book or store merchandise without reporting it.

[Q129] I have some pretty awful habits.

[Q130] I don’t gossip about other people’s business.

Describe yourself as you generally are now, not as you wish to be in the future. Describe yourself as you honestly see yourself, in relation to other people you know of the same sex as you are, and roughly your same age. So that you can describe yourself in an honest manner, your responses will be kept in absolute confidence. Indicate for each statement whether it is 1. Very Inaccurate, 2. Moderately Inaccurate, 3. Neither Accurate Nor Inaccurate, 4. Moderately Accurate, or 5. Very Accurate as a description of you. **[For Q131 - Q150: 6-item**

Likert scale; 1 - very inaccurate; 2 - moderately inaccurate; 3 - neither accurate nor inaccurate; 4 - moderately accurate; 5 - very accurate; 6 - prefer not to answer]

[Q131] Am the life of the party.

[Q132] Sympathize with others' feelings.

[Q133] Get chores done right away.

[Q134] Have frequent mood swings.

[Q135] Have a vivid imagination.

[Q136] Don't talk a lot.

[Q137] Am not interested in other people's problems.

[Q138] Often forget to put things back in their proper place.

[Q139] Am relaxed most of the time.

[Q140] Am not interested in abstract ideas.

[Q141] Talk to a lot of different people at parties.

[Q142] Feel others' emotions.

[Q143] Like order.

[Q144] Get upset easily.

[Q145] Have difficulty understanding abstract ideas.

[Q146] Keep in the background.

[Q147] Am not really interested in others.

[Q148] Make a mess of things.

[Q149] Seldom feel blue.

[Q150] Do not have a good imagination.

[Q151] We now ask you for your willingness to act in a certain way. Please again indicate your answer on a scale from 0 to 10. A 0 means “completely unwilling to do so,” and a 10 means “very willing to do so.”

How willing are you to give to good causes without expecting anything in return? **[Likert scale]**

[Q152] Imagine the following situation: Today you unexpectedly received 1,600 pounds. How much of this amount would you donate to a good cause? **[Numeric input]**

[Depending on the treatment allocation subjects saw one of the following two IOS scales for a stranger]

Please note: All questions below refer to a stranger.

[Q153a] In the following figure we ask you to consider which of these pairs of circles best represents your relationship with a stranger. In the figure “X” serves as a placeholder for a stranger, that is, you should think of “X” being the stranger. By selecting the appropriate number please indicate to what extent you and a stranger are connected. **[Numeric input]**

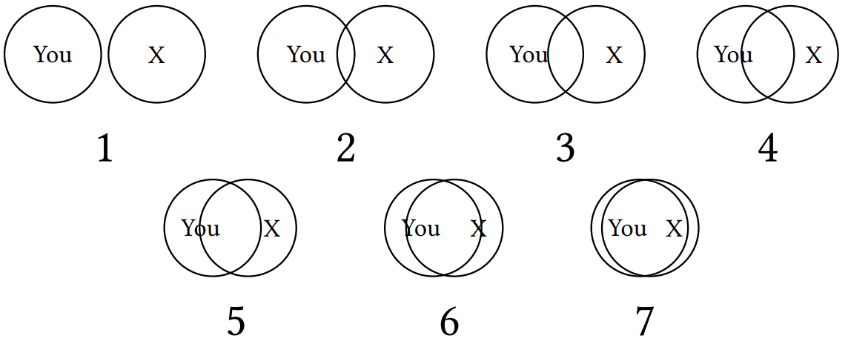

OR

“Once you move the slider below, a pair of circles will appear in the box. The position of the slider will determine the extent to which the circles overlap. When the slider is all the way to the left, the circles will look like this 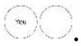*. When the slider is near the middle, the circles look like this* 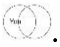*. With it all the way to the right the circles look like this* 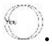*. You should interpret the degree of overlap as representing the relationship between you and a stranger. In the figure “X” serves as a placeholder for a stranger, that is, you should*

think of "X" being the stranger.

Please position the slider so that the circles indicate to what extent you and a stranger are connected."

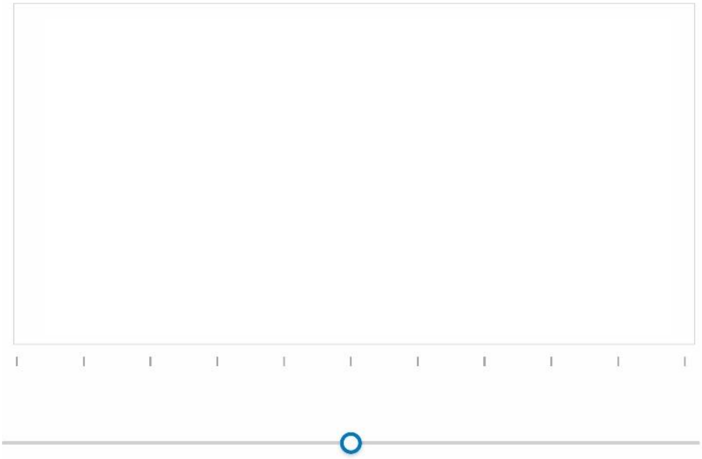

[Q154] Please, select the appropriate number below to indicate to what extent you would use the term "WE" to characterize you and a stranger. **[8-item Likert scale; 1 - Not at all; 7 - very much so; 8 - prefer not to answer]**

Thank you!

You're almost done, just answer these two questions and the study is done.

[Q155] To what extent have you participated in other studies involving similar questionnaires on Prolific before taking this study? Take a guess if you are not sure. **[5-item Likert scale; 1 - never; 2 - 1-10; 3 - 11-20; 4 - 21-50; 5 - more than 50]**

[Q156] What is your nationality?

- UK
- Other **[Text input]**
